# S1PR1 signaling attenuates apoptosis of retinal ganglion cells via modulation of cJun/Bim cascade and Bad phosphorylation in a mouse model of glaucoma

**DOI:** 10.1096/fj.202201346R

**Published:** 2022-12-15

**Authors:** Devaraj Basavarajappa, Vivek Gupta, Roshana Vander Wall, Veer Gupta, Nitin Chitranshi, Seyed Shahab Oddin Mirshahvaladi, Viswanthram Palanivel, Yuyi You, Mehdi Mirzaei, Alexander Klistorner, Stuart L. Graham

**Affiliations:** ^1^ Macquarie Medical School, Faculty of Medicine, Health and Human Sciences Macquarie University North Ryde Sydney New South Wales Australia; ^2^ School of Medicine Deakin University Geelong Victoria Australia

**Keywords:** apoptosis, cell signaling, glaucoma, intraocular pressure, neurodegeneration, neuroprotection, siponimod, sphingosine‐1‐phosphate

## Abstract

Glaucoma is a complex neurodegenerative disease characterized by optic nerve damage and apoptotic retinal ganglion cell (RGC) death, and is the leading cause of irreversible blindness worldwide. Among the sphingosine 1‐phosphate receptors (S1PRs) family, S1PR1 is a highly expressed subtype in the central nervous system and has gained rapid attention as an important mediator of pathophysiological processes in the brain and the retina. Our recent study showed that mice treated orally with siponimod drug exerted neuroprotection via modulation of neuronal S1PR1 in experimental glaucoma. This study identified the molecular signaling pathway modulated by S1PR1 activation with siponimod treatment in RGCs in glaucomatous injury. We investigated the critical neuroprotective signaling pathway in vivo using mice deleted for S1PR1 in RGCs. Our results showed marked upregulation of the apoptotic pathway was associated with decreased Akt and Erk1/2 activation levels in the retina in glaucoma conditions. Activation of S1PR1 with siponimod treatment significantly increased neuroprotective Akt and Erk1/2 activation and attenuated the apoptotic signaling via suppression of c‐Jun/Bim cascade and by increasing Bad phosphorylation. Conversely, deletion of S1PR1 in RGCs significantly increased the apoptotic cells in the ganglion cell layer in glaucoma and diminished the neuroprotective effects of siponimod treatment on Akt/Erk1/2 activation, c‐Jun/Bim cascade, and Bad phosphorylation. Our data demonstrated that activation of S1PR1 in RGCs induces crucial neuroprotective signaling that suppresses the proapoptotic c‐Jun/Bim cascade and increases antiapoptotic Bad phosphorylation. Our findings suggest that S1PR1 is a potential therapeutic target for neuroprotection of RGCs in glaucoma.

**Abbreviations**
AAV: adeno‐associated virus
CNS: central nervous system
DAPI: 4′,6‐diamidino‐2‐phenylindole
GCL: ganglion cell layer
GFP: green fluorescent protein
hSyn1: human synapsin 1
IF: immunofluorescence
IOP: intraocular pressure
NeuN: neuronal nuclear antigen
RGC: retinal ganglion cell
S1PR: sphingosine‐1‐phosphate receptor
VG: viral genomes
WB: western blot

## 1 INTRODUCTION

Glaucoma is a complex, multifactorial neurodegenerative disease that causes optic nerve damage, and progressive loss of retinal ganglion cells (RGCs) in the inner retina, eventually leading to irreversible vision loss.^1^ Elevated intraocular pressure (IOP) is one of the major established risk factors contributing to glaucoma development and progression.^2^, ^3^ Surgery and pharmacological medications that mainly aim to lower the elevated IOP are not adequate to prevent RGC loss, and in many patients, the vision continues to decline even after IOP management.^4^, ^5^ The exact mechanism that contributes to glaucoma pathophysiology is currently not well defined. The death of RGCs in glaucoma is predominantly mediated by apoptosis programs that occur through a complex sequence of mechanisms, including disruption of neurotrophic factors, oxidative stress, mitochondrial dysfunction, endoplasmic reticulum stress, excitotoxicity, ischemia, and glial activation.^6^, ^7^ Identifying the underlying molecular pathways of RGC survival in glaucoma could lead to the development of new protective therapeutics. In this regard, the neuroprotective strategies identify and target the potential molecular pathways intended to delay or prevent the death of RGCs by mitigating the proapoptotic process.^8^, ^9^

Bioactive sphingolipids—ceramide, sphingosine, and sphingosine‐1‐phosphate (S1P)—are signaling molecules that regulate diverse cellular functions, including cell survival, growth, migration, and differentiation in the retina and the brain.^10^, ^11^, ^12^ Particularly, S1P activates five G‐protein‐coupled receptors termed S1PR1‐5 and are implicated in the pathogenesis of different neurodegenerative diseases in the central nervous system (CNS).^13^ S1PR1 is the most widely expressed receptor subtype among all five receptors in the CNS and has gained increasing attention as a mediator of cellular survival and inflammation in retinal cells.^14^ S1PR1 is exclusively coupled with Gi protein that activates multiple intracellular downstream molecules, including phosphatidylinositol‐3‐kinase (PI3K)/protein kinase B (Akt), phospholipase C (PLC), adenylyl cyclase (AC), and Erk/MAP kinase pathways.^15^, ^16^ The PI3K/Akt pathway plays a crucial role in mediating the signals that participate in neuronal cell growth, proliferation, and survival.^17^ The activation of PI3K/Akt/Erk signaling pathways has been demonstrated to suppress the apoptotic pathway and promote neuronal cell survival during injuries.^17^, ^18^, ^19^, ^20^

Siponimod (BAF312) is an S1P structural analog that modulates S1P receptor signaling, binds specifically to S1P1 and S1P5 receptors, and is an oral immunosuppressive drug approved for the management of multiple sclerosis.^21^ The immunomodulatory properties of siponimod impair the autoreactive lymphocytes egress from the lymph nodes and thus prevent their infiltration into the CNS and reduce inflammation.^22^ In addition, siponimod could cross the blood–brain barrier (BBB) and exhibit neuroprotective effects in various preclinical models by directly interacting with the CNS‐resident cells.^23^, ^24^, ^25^ We recently showed that siponimod treatment exerted neuroprotection against glaucoma injury. Further, the study revealed that neuronal S1PR1 ablation enhanced neuronal degeneration and siponimod mediates its protective effects through neuronal S1PR1 in experimental glaucoma.^26^ However, the potential implication of S1PR1‐mediated signaling in protecting RGCs against glaucomatous injury‐induced apoptotic pathways remains not understood. In this study, we have identified the molecular signaling mechanism behind those protective effects of siponimod and defined the role of S1PR1 in RGCs in inducing protective survival signaling. Our results show that the neuronal S1PR1 regulates Akt/Erk‐cJun/Bim cascade and Bad phosphorylation to induce neuroprotective effects against RGCs apoptotic death in high IOP conditions.

## 2 MATERIALS AND METHODS

### 2.1 Experimental animals

All animal experiments in this study were performed in accordance with the Australian Code of Practice for the Care and Use of Animals for Scientific Purposes and the guidelines of the ARVO (the Association for Research in Vision and Ophthalmology) Statement for the Use of Animals in Ophthalmic and Vision Research, and all procedures were approved by Macquarie University Animal Ethics Committee. The transgenic S1PR1^*flox/flox*^ mice (B6.129S6(FVB)‐S1pr1^tm2.1Rlp^/J; RRID: IMSR_JAX:019141) were obtained from the Jackson Laboratory (Bar Harbor, ME, USA) and bred at the Animal Care Facility of Macquarie University under specific pathogen‐free conditions. The animals recruited (a total of 80 mice) for different experimental groups were maintained in an air‐conditioned room with a controlled temperature (21–28°C). All the animals had fixed daily 12‐h light/dark cycles. Siponimod (BAF312) was obtained from Novartis (Basel, Switzerland) and administered orally to the recipient mice through diet as described previously. A dose of 10 mg/kg siponimod was used as an optimized dose (ad libitum), and 3–4 mice were kept in each cage.^27^ The animals were randomly divided into four groups (10 mice in each group): (1) control (sham, normal IOP) group, (2) control (sham, normal IOP) group with siponimod treatment, (3) high IOP subjected untreated group, and (4) high IOP subjected group with siponimod treatment.

### 2.2 Generation of neuronal S1PR1‐deficient mice

Neuronal‐specific deletion of S1PR1 was carried out in S1PR1^*flox/flox*^ mice by administering Cre recombinase through adeno‐associated viruses (AAVs, stereotype AAV‐PHP.eB) as described previously.^26^ AAV‐PHP.eB (Vector Biolabs, USA) expressing the Cre recombinase under neuron‐specific promoter hSyn1 (AAV‐PHP.eB‐Syn1‐GFP‐Cre and AAV‐PHP.eB‐Syn1‐GFP as control) were delivered systemically to S1PR1^*flox/flox*^ adult mice via tail vein injections. The mice subjected to the IOP model received AAV injections 1 week before induction of elevated IOP. Briefly, the mice were anesthetized with 2%–5% isoflurane in oxygen, and then animals were maintained on 1%–3% isoflurane in oxygen (0.6–1 L/min flow of oxygen) on a heating pad during the injection procedure. The mice tails were warmed in 40°C water for 1 min and swabbed with 70% alcohol pads before injections. The viral suspension of different doses (1.5 × 10^9^, 1.5 × 10^10^ and 1.5 × 10^11^ vg) in 50 μl was slowly delivered into a lateral tail vein using a 33G needle attached to the Hamilton syringe.

### 2.3 Animal model of glaucoma induced by intraocular microbead injections

A chronic experimental glaucoma model was established by inducing elevated IOP in mice by microbeads (10 μm diameter FluoSpheres; Invitrogen, USA) injections into the anterior chamber of the eyes as described previously.^28^, ^29^, ^30^, ^31^ Briefly, mice were anesthetized with ketamine (75 mg/kg) (Provet, NSW, Australia) and medetomidine (0.5 mg/kg) (Troy Laboratories NSW, Australia) intraperitoneal injections and placed on a warming pad. After anesthesia, the pupils were dilated with topical tropicamide 1%, and proxymetacaine 0.5% (Alcon Laboratories NSW, Australia) eye drops were applied as a topical anesthetic. Intraocular injections were performed (2 μl; 3.6 × 10^6^ microbeads/ml) using a Hamilton syringe connected to a disposable 33G needle (TSK Laboratory, Tochigi, Japan). The cornea was punctured gently, and the needle was inserted tangentially beneath the corneal surface and 2 μl of microbeads were slowly released into the anterior chamber. All intracameral procedures were performed under an operating microscope (OPMI Vario S88, Carl Zeiss, Oberkochen, Germany) with care taken to avoid any needle contact with the iris or lens. One of the eyes was randomly selected for injection, and an equivalent volume of sterile PBS was injected into the eyes of control (sham) animals (normal‐IOP). At the end of the procedure, anesthesia was reversed with a subcutaneous injection of atipamezole (0.75 mg/kg), and 0.3% ciprofloxacin drops (Ciloxan; Alcon Laboratories Pty Ltd, NSW, Australia) and 0.1% dexamethasone eye drops (Maxidex, Novartis Pharmaceuticals Australia, NSW, Australia) were applied to the injected eyes. Lacrilube (Allergan Australia Pty Ltd, Gordon, NSW, Australia) was then applied to the cornea. Intraocular pressure was monitored weekly after microbead injections and further injections were given to the eyes where IOP was observed to be below 20 mmHg. Mice eyes with sustained high IOP elevation were only included in this study, and animals with any kind of damage to the eyes were excluded. The weekly IOP measurements were carried out using an iCare TonoLab rebound tonometer (Icare Tonovet, Helsinki, Finland) as described previously.^29^, ^32^

### 2.4 Apoptotic assay (TUNEL staining)

To assess cellular apoptotic changes in the retinas, terminal deoxynucleotidyl transferase‐mediated dUTP‐biotin nick end labeling (TUNEL) staining was performed on paraffin‐embedded retinal sections using DeadEnd Fluorometric TUNEL System kit (Promega) as described previously.^29^, ^32^ Briefly, parafilm retinal sections were deparaffinized with xylene and rehydrated through a series of decreasing concentrations of ethanol followed by a 15‐min fixation in 4% PFA solution and washed with PBS. The rehydrated sections were permeabilized with proteinase K (20 μg/ml), washed, and refixed in 4% PFA for 5 min. The sections were then washed with PBS and incubated with a TUNEL reaction mixture (containing equilibration buffer, nucleotide mix, and rTdT enzyme) at 37°C for 1 h in the dark. A plastic coverslip was placed over the slides to avoid tissue from getting dry and for even distribution of the reaction mix. Following incubation, the slides (without plastic coverslip) were immersed in 2× saline‐sodium citrate (SSC) buffer for 15 min to stop the reaction. The slides were then washed with PBS and mounted with prolonged anti‐fade mounting media with Prolong 4′,6‐diamidino‐2‐phenylindole (DAPI; Life Technologies, Eugene, OR, USA). The apoptotic cell staining was examined using epifluorescence microscopy (ZEISS Axio Imager, Carl Zeiss, Oberkochen, Germany), and TUNEL‐positive cells were quantified over 500 μM (100–600 μM and 600–1200 μM regions from the edge of the optic disk) from the immunofluorescence images (*n* = 5 in each group).

### 2.5 Immunofluorescence (IF) staining

Mice were killed at specified time points and tissues were harvested from the mice after perfused transcardially with PBS and freshly prepared 4% paraformaldehyde (PFA). The eyeballs were marked with tissue marking dye to maintain the similar orientation during tissue embedding. The animal eyes were then fixed for 1 h in 4% PFA and washed with PBS. Post‐fixed eyes were processed via an automatic tissue processor (ASP200S, Leica, Nussloch, Germany) before embedding in paraffin or postfixed tissues were embedded in optimal cutting temperature compound (OCT) (Sakura Finetek, Torrance, CA, USA) in dry ice as described previously.^30^, ^31^, ^32^ Tissue marking was used to ensure similar orientation for all eyes during tissue embedding, and immunofluorescence assessment was done on cryosections or deparaffinized sections. A total quantity of 7μm thick sagittal retinal sections were treated with a blocking buffer containing 0.3% Triton X‐100 and 5% serum of the secondary species (donkey, Sigma‐Aldrich, St. Louis, MO, USA) in PBS for 2 h at room temperature. The sections were then incubated with the indicated primary antibodies overnight at 4°C prepared in antibody dilution buffer (1 × PBS/2% BSA/0.3% Triton X‐100). The following primary antibodies were used for immunofluorescence staining: anti‐GFP (green fluoresce protein) (1:500; Abcam, ab290), anti‐NeuN (1:1000; Abcam, ab104224), and anti‐Bim (1:500; Cell Signaling, 2933). After primary antibody incubations, the tissue sections were washed three times with PBS and incubated with appropriate secondary antibodies (donkey anti‐rabbit Cy3 or donkey anti‐mouse Alexa Fluor 488; Jackson ImmunoResearch Labs) for 60 min at room temperature. The sections were washed three times with PBS and mounted with anti‐fade mounting media with Prolong DAPI (Life Technologies, Eugene, OR, USA). All images were acquired using a Zeiss fluorescence microscope (ZEISS Axio Imager, Carl Zeiss, Oberkochen, Germany). To assess the percentage of AAV transduction, GFP^+^/total NeuN cells were measured in three cross‐sections per eye in each group of mice using ImageJ software (ImageJ, v 1.52; NIH, Bethesda, MD, USA).

### 2.6 Western blot (WB) analysis

Retinal tissue harvested from enucleated eyes were span frozen in liquid nitrogen and subsequently resuspended in ice‐cold lysis buffer (20 mM Tris–HCl, pH 8.0, 1% Triton X‐100, 2 mM EDTA, 2 mM PMSF, 100 mM NaCl, 1 mM Na_3_VO_4_, 0.1% SDS, and complete protease inhibitor cocktail). Retinal tissue samples were homogenized by sonication and centrifuged at 10 000× *g* for 10 min at 4°C to collect protein supernatants. The protein concentration of the obtained lysates was quantified by Micro BCA assay (Thermo Fisher Scientific, MA, USA). Proteins (about 20 μg) were subjected to SDS‐PAGE and transferred to nitrocellulose membrane by electroblotting (Invitrogen iBlot2, Thermo Fisher Scientific, MA, USA) as described previously.^33^, ^34^, ^35^Following protein transfer, the membranes were washed with TTBS (20 mM Tris–HCl pH 7.4, 100 mM NaCl, and 0.1% Tween 20) and blocked with nonfat dry milkpowder (5%) in TTBS for 1 h at room temperature. Blots were then incubated with primary antibodies at 4°C overnight. The primary antibodies with concentration used were: anti‐phospho‐Akt (Ser473) (1:1000; XP 4060); anti‐Akt (1:1000; 4685); anti‐phospho‐p44/42 MAPK (Erk1/2) (Thr202/Tyr204) (1:1000; 4376); anti‐p44/42 MAPK (Erk1/2) (1:1000; 4695); anti‐Bcl2 (1:1000; 2876); anti‐Bim (1:1000; 2933); anti‐pBad (Ser136) (1;1000; 9295); anti‐Bad (1;1000; 9292); anti‐phospho‐c‐Jun (Ser63) (1:1000; 9261); and anti‐c‐Jun (60A8) (1:1000; 9165) were from Cell Signaling. Anti‐GFP (1:1000; ab290), anti‐S1P1/EDG1 (1:1000; ab11424), anti‐Bax (1:1000; ab32503), and anti‐β‐actin (1:5000; ab6276) were from Abcam. Following primary antibody incubations, the membranes were washed four times (5 min each) with TTBS and incubated for 1 h at room temperature with secondary antibody conjugated with horseradish peroxidase (anti‐rabbit 1:5000, and anti‐mouse 1:5000, Jackson ImmunoResearch Labs). After four times of washing, the protein bands were detected by enhanced chemiluminescence (Super Signal West Femto Maximum Sensitive Substrate; Thermo Fisher Scientific) according to the manufacturer's instructions, and images were captured with a Bio‐Rad ChemiDoc^MP^ Imaging system (Bio‐Rad Laboratories, Inc., Hercules, CA, USA). The mean densitometric analysis of the protein band intensities was performed after the relative expression of the target proteins normalized to β‐actin using ImageJ software.

### 2.7 Statistical analysis

Statistical analysis of all data obtained in this study was performed using GraphPad Prism 8 software (GraphPad Software Inc., San Diego, CA, USA). The animal tissues from the four experimental groups were utilized to analyze the retinal apoptotic changes by TUNEL assay, western blotting of target proteins, and immunofluorescence staining of the retinal tissues. The number of samples (*n*) indicated in each figure represents the tissues from the different animals of the same group. Comparisons between the groups were performed by one‐way ANOVA analysis followed by Tukey's multiple comparisons test. All the data are presented as mean ± standard deviation of the mean (SD) for given n sizes and a *p*‐value < .05 was considered statistically significant for data analysis.

## 3 RESULTS

### 3.1 Efficient delivery of AAV‐PHP.eB generates S1PR1 deletion in RGCs

**FIGURE 1:**
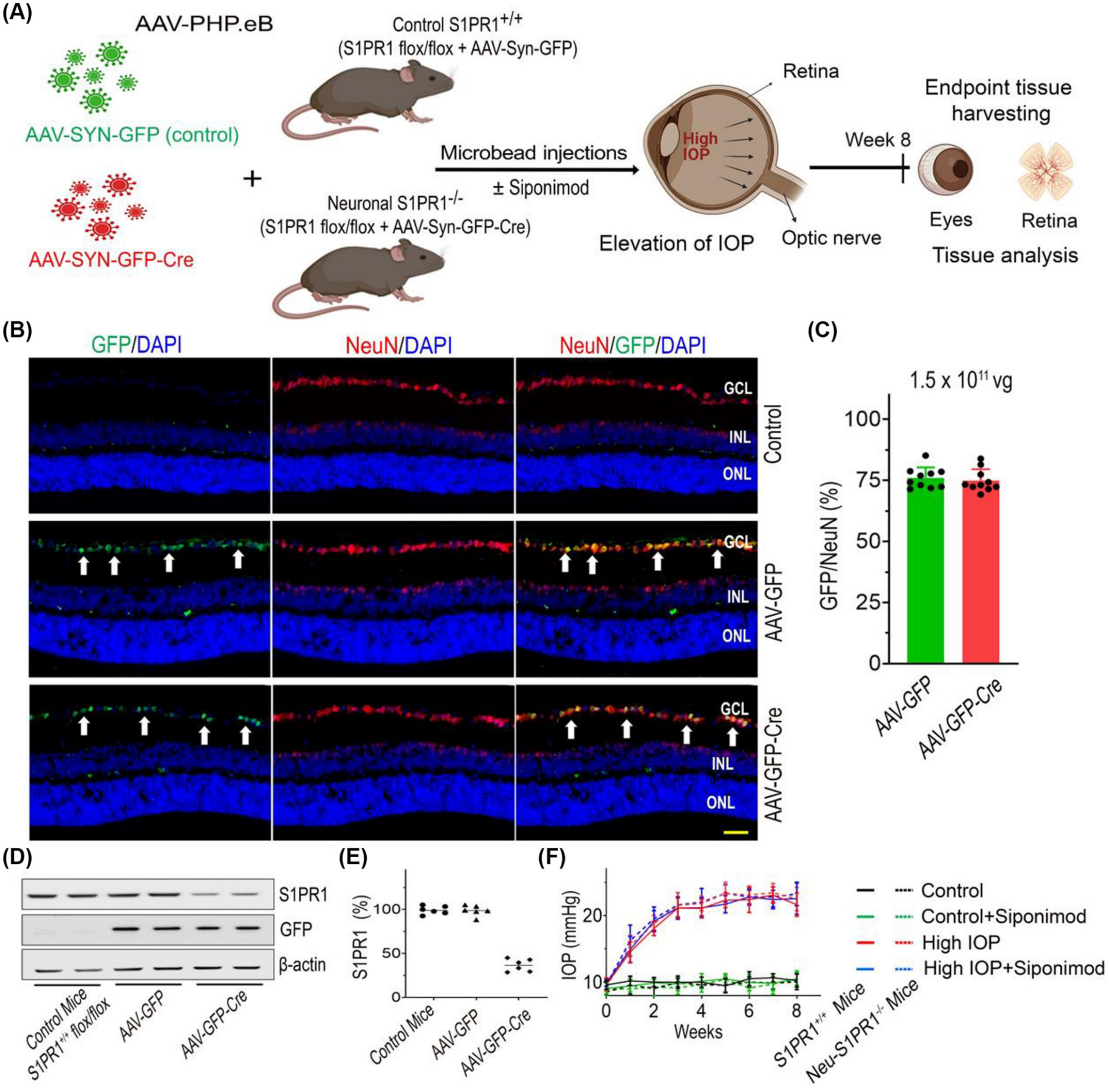
Generation of neuronal‐specific S1PR1‐deleted mice using AAV‐PHP.eB‐Syn1‐GFP‐Cre in S1PR1^*flox/flox*^ transgenic mice and induction of chronic elevated intraocular pressure (IOP) in mice. (A) Schematic representation of delivery of AAV‐PHP.eB vector constructs expressing Cre recombinase under neuron‐specific promoter, Syn1, via tail vein injection into S1PR1^*flox/flox*^ mice, and depiction of the experimental model of glaucoma design and analysis of the tissues. (B) Immunofluorescence images of the eye sections (representative: GCL, ganglion cell layer; INL, inner nuclear layer; ONL, outer nuclear layer) stained with GFP (green) and NeuN (red) showing the efficient infection of RGCs with AAVs (arrows indicate the expression and colocalization of GFP expression in RGCs in the GCL, Scale bar = 50 μM) and (C) transduction efficiency (GFP^+^ cells/total NeuN cells) of viral vectors after 2 months of delivery the mice that received 1.5 × 10^11^ vg (*n* = 10 per group). (D) Analysis of the retinal tissues for GFP and S1PR1 expression by western blotting, and (E) densitometric quantification of western blot band intensities for S1PR1 deletion after normalizing to β‐actin (*n* = 5 per group). (F) Induction of chronic elevation of IOP in different groups' mice eyes by intracameral microbead injections for 8 weeks (*n* = 10 mice per group).

S1PR1 is expressed by a variety of neurons in the CNS, including RGCs^36^, ^37^, ^38^ and the global deletion of S1PR1 gene was found to be embryonically lethal.^39^ Therefore, to generate neuronal‐specific deletion S1PR1 mice, we delivered Cre recombinase through AAV‐PHP.eB vector into the adult S1PR1^*flox/flox*^ mice. AAV‐PHP.eB is an engineered novel variant of AAV9 and, when delivered systemically by intravenous injections demonstrated widespread transduction of both the brain and retina.^40^, ^41^ Cre‐recombinase integrated into AAV‐PHP.eB vector under neuron‐specific promoter, Syn1, was delivered via tail vein injection into adult S1PR1^*flox/flox*^ mice before subjecting the mice to experimental model of glaucoma (Figure 1A). We first delivered different genomic copies of viral vectors to determine the transduction efficacy of AAVs. The transduction assessed by costaining of retinal sections with GFP and NeuN showed colocalization of GFP with NeuN. The transduction efficiency measured as the ratio of GFP^+^ cells to the total NeuN cells showed around 43% and 62% for the mice injected with 1.5 × 10^9^ and 1.5 × 10^10^ vg of viral vectors, respectively, after 2 months of vector delivery (Figure S1). At 1.5 × 10^11^ vg dose, the transduction efficiency 76.03% ± 4.28% for GFP control (AAV‐GFP) and 74.89% ± 5.63% for Cre (AAV‐GFP‐Cre) AAVs were achieved (Figure 1B,C), and this dose was chosen for the mice subjected to microbead injections. GFP expression analyzed at different time points revealed the maximum expression after 1 month of viral vector delivery (Figure S2). WB densitometric quantification further showed that the S1PR1 expression in the retina lysates was decreased by 63.49% ± 7.05% in AAV‐GFP‐Cre‐injected mice compared to the control mice (AAV‐GFP) (Figure 1D,E).

### 3.2 S1PR1 activation with siponimod treatment protects high IOP‐induced RGCs apoptosis

**FIGURE 2:**
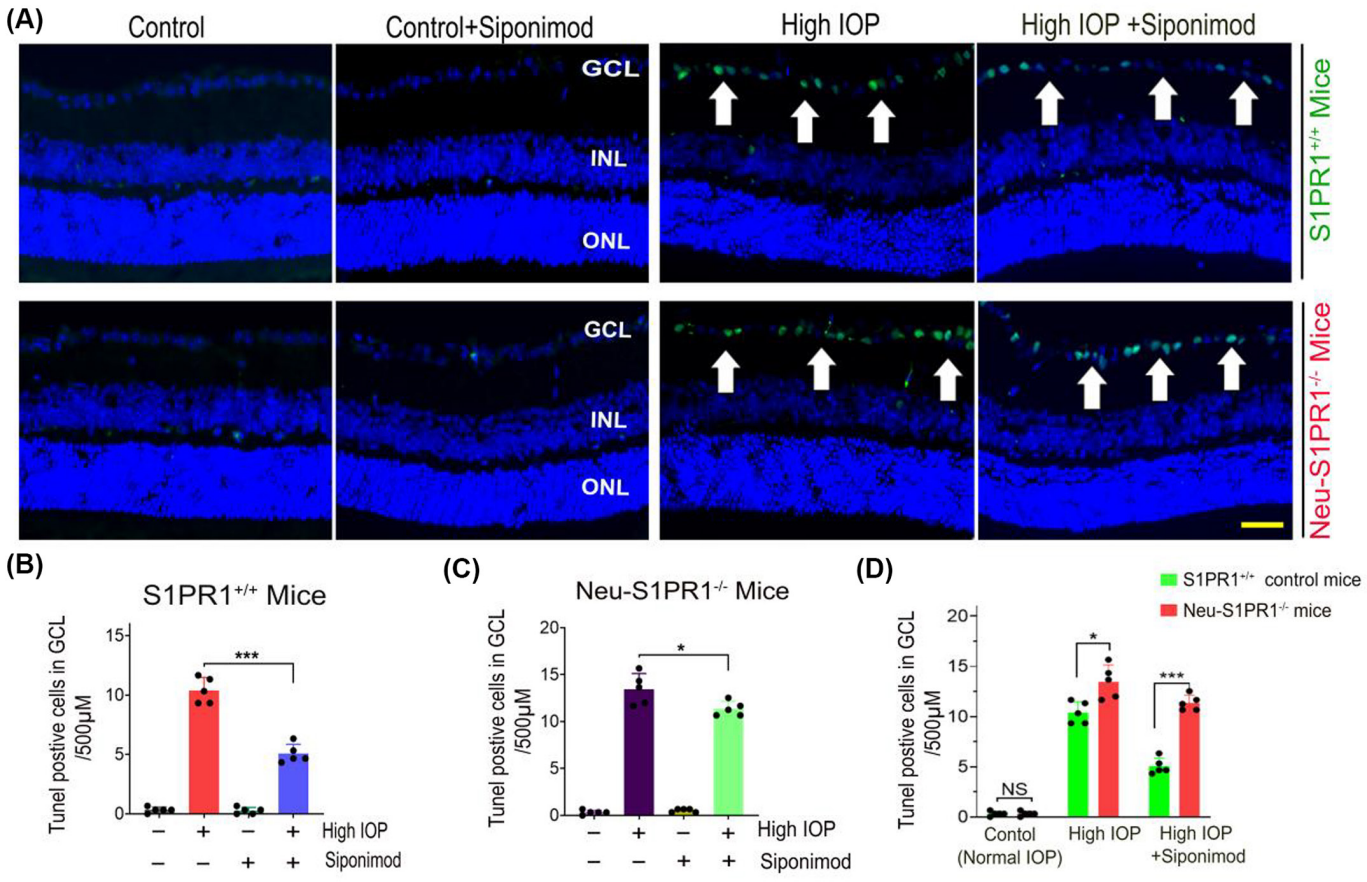
Deletion of S1PR1 in RGCs increases apoptotic changes in the ganglion cell layer (GCL) and diminishes the protective effects of siponimod in an experimental model of glaucoma. (A) Analysis of TUNEL apoptotic changes assessed in 100–600 μM region from the edge of optic disk in the retinal sections (arrows indicate changes in the TUNEL staining in the GCL) after 8 weeks of elevated IOP induction in different mice groups (representative image of TUNEL staining [green] and nuclear staining DAPI [blue]), Scale bar = 50 μM; GCL, ganglion cell layer; INL, inner nuclear layer; ONL, outer nuclear layer). (B) Quantification of TUNEL‐positive cells in the GCL of retinas from the S1PR1^+/+^ control mice group and (C) Neu‐S1PR1^−/−^ mice group. (D) Comparison of the TUNEL‐positive cells in the GCL among different mice groups showed S1PR1 deletion in RGCs significantly increased the apoptotic cells and diminished the protective effects of siponimod in elevated IOP conditions (NS, not significant, *p < .05, ***p < .001, one‐way ANOVA analysis with Tukey's multiple comparisons test, *n* = 5 per group).

The death of RGCs in glaucomatous conditions occurs primarily by the apoptotic pathway.^42^ We analyzed retinal sections for apoptotic changes to investigate the role of neuronal S1PR1 signaling in neuroprotective mechanisms in glaucomatous conditions. Mice injected with AAV‐GFP referred to as S1PR1^+/+^ control mice and AAV‐GFP‐Cre mice referred to as Neu‐S1PR1^−/−^ mice were subjected to intraocular microbead injections to induce elevated IOPs for 2 months. Mice groups with intracameral microbeads injections exhibited elevated IOPs (20–25 mmHg) and the IOPs of the siponimod‐treated mice were comparable with the untreated control mice for eight weeks (Figure 1F). The TUNEL apoptotic cells were assessed at a distance of 500 μM on retinal sections in two different regions (100–600 μM and 600–1200 μM from the edge of the optic disk). Eight weeks of chronic high IOP elevation significantly increased the apoptotic cells in the GCL layer (*p* < .0001) in both the regions of the retina with relatively more TUNEL‐positive cells observed in the region near to optic disc (100–600 μM) compared to the far region (600–1200 μM). The siponimod treatment significantly reduced the TUNEL‐positive cells compared to untreated mice in high IOP conditions (*p* < .001) in the S1PR1^+/+^ control mice group in both regions of the retina (Figures 2A,B and S3). Deletion of S1PR1 in RGCs significantly increased the apoptotic cells in the GCL compared to S1PR1^+/+^ control mice in high IOP conditions (*p* < .05). Further, in the Neu‐S1PR1^−/−^ mice group, the protective effects of siponimod treatment on RGCs apoptotic changes were decreased drastically in high IOP conditions (Figures 2C and S3C). A significantly increased number of TUNEL‐positive cells (*p* < .001) was observed in Neu‐S1PR1^−/−^ mice compared to the S1PR1^+/+^ control mice with siponimod treatment in high IOP conditions (Figures 2D and S3D). These results suggested that S1PR1 signaling in RGCs is neuroprotective in glaucomatous injury conditions.

**FIGURE 3:**
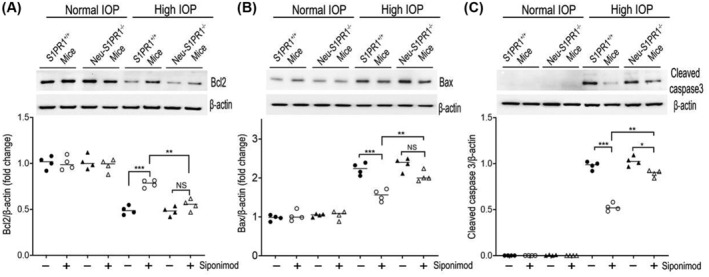
Siponimod treatment restores the apoptotic marker changes in the retina in high IOP conditions and S1PR1 deletion in RGCs reduces the antiapoptotic effects of siponimod. Effects of siponimod treatment and RGC‐specific deletion of S1PR1 on the apoptotic marker changes in the retina were evaluated by western blotting of the retinal tissues following 8 weeks of chronic elevated IOP. (A) Representative immunoblots and densitometric analysis for the levels of Bcl2 expression, (B) Bax expression, and (C) the levels of cleaved casapase‐3. Densitometric quantification of western blot band intensities were determined after normalizing to β‐actin. (NS, not significant, *p < .05, **p < .01, ***p < .001, one‐way ANOVA analysis with Tukey's multiple comparisons test, *n* = 4 per group).

We next evaluated the changes in the expression of apoptotic pathway proteins, Bax, Bcl2, and active caspase 3.^43^ As members of the Bcl2 protein family, antiapoptotic factors Bcl‐2 and proapoptotic factor Bax play important roles in RGCs apoptosis, and their expressions were demonstrated to be alerted in glaucomatous injury.^44^ The intrinsic initiation of the proapoptotic pathway through Bax results in the activation of caspase 3 which directly induces RGC death. Thus, we examined how these apoptosis‐related protein expressions are changed with S1PR1 deletion in RGCs and siponimod treatment conditions by western blot analysis of mice retinas that were subjected to 8 weeks of chronic high IOP. The expression of the antiapoptotic protein Bcl‐2 was decreased by 1.82 ± 0.20‐fold (Figure 3A), and the proapoptotic protein Bax was increased significantly by 2.23 ± 0.15‐fold (Figure 3B) with the upregulation of cleaved caspase‐3 levels (Figure 3C) in high IOP conditions. Siponimod treatment showed protective effects by restoring these expression changes to a significant extent in S1PR1^+/+^ control mice (*p* < .001). However, S1PR1 deletion in RGCs abolished these protective effects of siponimod in glaucoma injury. The protective effects of siponimod on the restoration of apoptotic pathway proteins Bcl2, Bax, and activated caspase 3 were diminished significantly in the Neu‐S1PR1^−/−^ mice compared to the S1PR1^+/+^ control mice group (*p* < .01) in high IOP conditions (Figure 3A–C). These results indicated that neural S1PR1 signaling protects the RGCs death by suppressing the proapoptotic pathway in experimental glaucomatous injury conditions.

### 3.3 S1PR1 deletion in RGCs attenuates siponimod‐induced Akt/Erk1/2 activation

**FIGURE 4:**
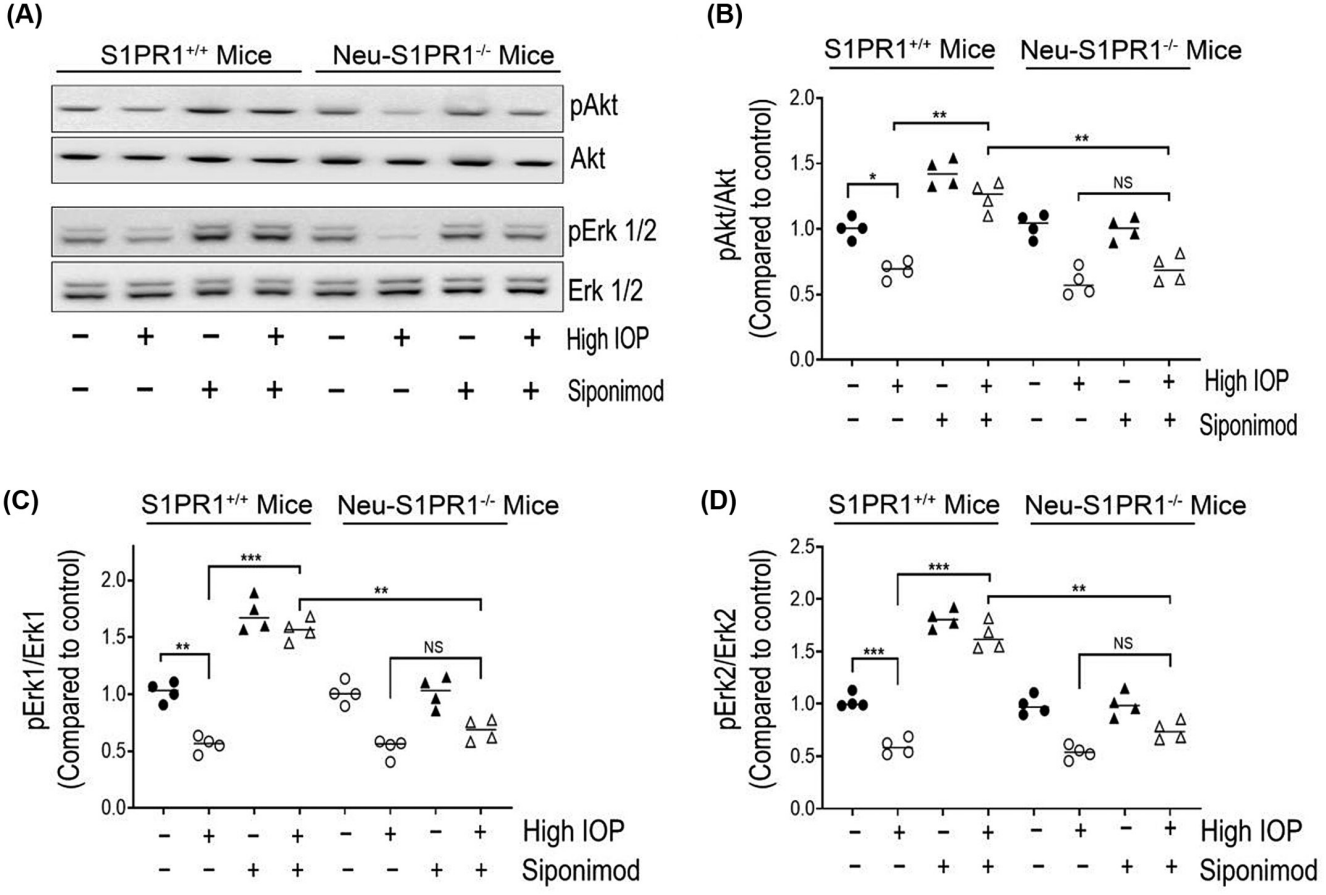
Siponimod treatment‐mediated upregulation of Akt and Erk 1/2 phosphorylation levels in the retina are reduced with S1PR1 deletion in RGCs. WB analysis of the effects of siponimod treatment and S1PR1 deletion in RGCs on the phosphorylation levels of Akt and Erk1/2 in the retinal tissues following 8 weeks of chronic elevated IOP. (A) Representative western blots showing the phosphorylation levels of Akt (phsopho‐S473 antibody) and Erk1/2 (Phospho‐p44/42 MAPK (Erk1/2) (Thr202/Tyr204) in the retinal tissues of the control S1PR1^+/+^ mice and Neu‐S1PR1^−/−^ (AAV‐GFP‐Cre) mice groups under normal and high IOP conditions. (B) Densitometric quantitative analysis of blot densities showing the reduced levels of phosphorylated Akt and (C and D) Erk 1/2 with siponimod upon S1PR1 deletion in RGCs (**p < .01, ***p < .001 one‐way ANOVA analysis with Tukey's multiple comparisons test, *n* = 4 per group).

We next explored the mechanism by which neuronal S1PR1 signaling protects RGC against glaucomatous‐induced cell death. It has been demonstrated that activation of pro‐survival Akt/Erk pathways protects neurons in degenerative conditions,^19^ and previously fingolimod treatments were shown to induce Akt/Erk phosphorylation through S1PR1.^45^ We, therefore, investigated the effect of siponimod treatments and neuronal deletion of S1PR1 on Akt/Erk activations in 8 weeks of high IOP‐exposed mice retinas. The western blot analysis of retinal lysates revealed a significant decrease (*p* < .05 for Akt and *p* < .01 for Erk) in Akt/Erk phosphorylation levels in high IOP retinas (Figure 4A). Siponimod‐treated mice retinas showed significantly upregulated Akt/Erk phosphorylation levels (*p* < .01 for Akt and *p* < .001 for Erk). However, upon S1PR1 deletion in RGCs, these siponimod‐induced Akt/Erk phosphorylation levels were significantly reduced (*p* < .01) (Figure 4B–D). These results indicated that activation of neuronal S1PR1 by siponimod upregulates Akt/Erk pathways in the retina to protect RGCs against high IOP‐induced apoptosis.

### 3.4 S1PR1‐mediated Akt/Erk1/2 activation downregulates c‐Jun/Bim pathway in RGCs in glaucomatous injury

**FIGURE 5:**
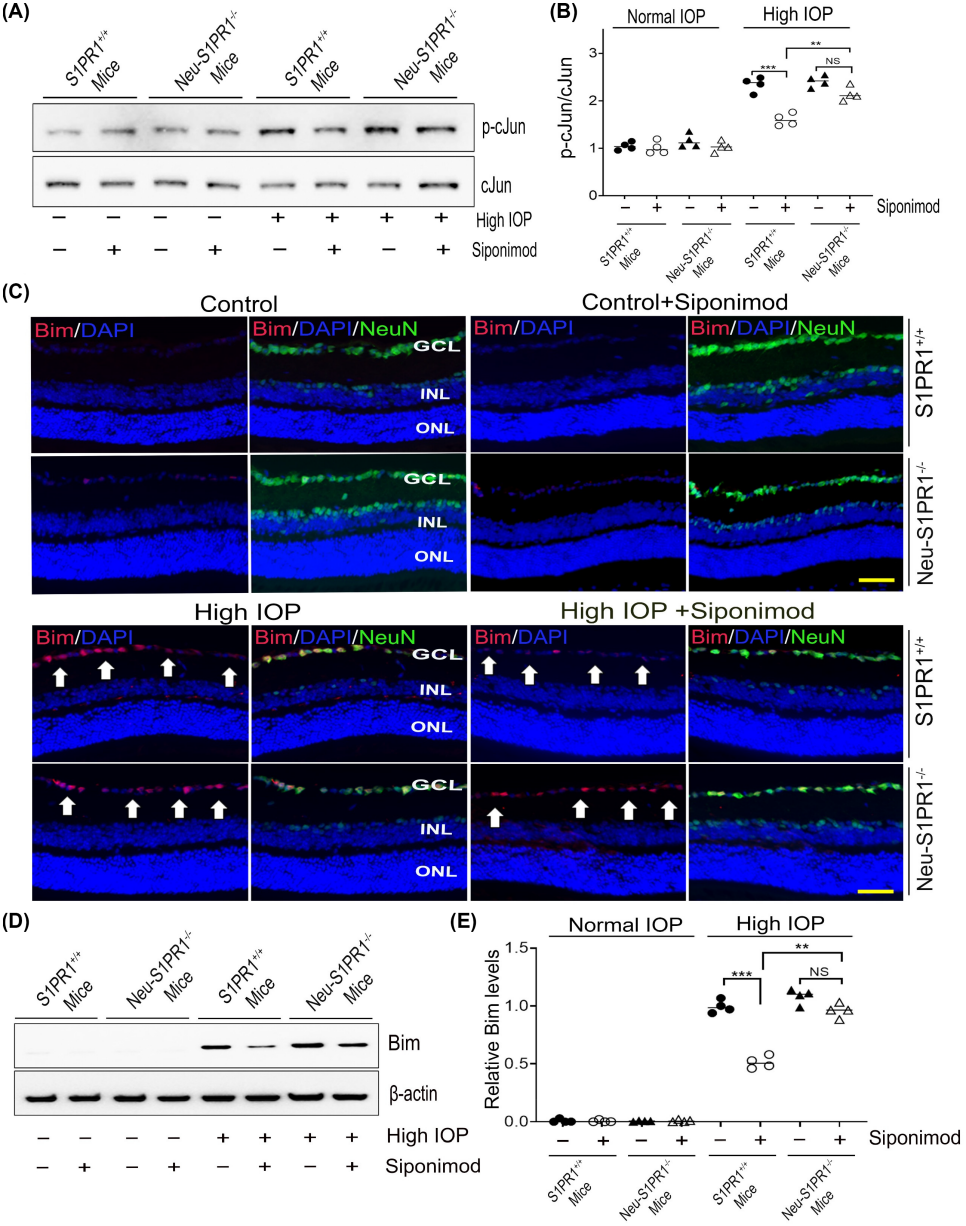
S1PR1 activation reduces the phosphorylation levels of c‐Jun and its downstream proapoptotic Bim expression in experimental glaucoma conditions. Activated transcription factor c‐Jun, which controls the expression of proapoptotic factor Bim in experimental glaucoma is downregulated with S1PR1 activation with siponimod treatment. (A) Analysis of phosphorylated levels of c‐Jun in the retinal samples from control S1PR1^+/+^ mice and Neu‐S1PR1^−/−^ (AAV‐GFP‐Cre) mice groups under normal and 8 weeks of high IOP conditions with/without siponimod treatment. (B) Densitometric quantitative analysis of phospho‐c‐Jun blot densities (p‐cJun/cJun) showing the significantly increased levels of activated c‐Jun in elevated IOP conditions was reduced with siponiomd treatment, and the deletion of S1PR1 in RGCs abolished the antiapoptotic effects of siponimod on the c‐Jun activation. (C) Representative immunofluorescence images of the eye sections stained with Bim (red) and NeuN (green) among different mice groups (Scale bar = 50 μM; GCL, ganglion cell layer; INL, inner nuclear layer; ONL, outer nuclear layer; arrows indicate changes in the relative expression of Bim in the GCL). (D) Representative immunoblots and (E) their quantitative densiometric analysis plot (after normalizing to β‐actin) showing the upregulation of Bim expression in high IOP conditions was significantly reduced with siponimod treatment and the deletion of S1PR1 in RGCs abolished the antiapoptotic effects of siponimod on the c‐Jun/Bim cascade (**p < .01, ***p < .001 one‐way ANOVA analysis with Tukey's multiple comparisons test, *n* = 4 per group).

Activation of c‐Jun in the intrinsic neuronal apoptotic pathway is commonly observed in neurodegenerative diseases, including glaucoma.^46^, ^47^ This activated transcription factor upregulates proapoptotic proteins (e.g., Bim, Bmf, Puma) that mediates neuronal cell death.^43^ The upregulated pro‐survival Akt/Erk1/2 has been demonstrated to be antiapoptotic through suppression of the JNK/c‐Jun pathway.^48^, ^49^ We investigated whether the S1PR1 signaling‐induced activation of Akt/Erk mediates the antiapoptotic effect through suppression of upregulated c‐Jun pathway. Western blot analysis of the retina lysates demonstrated marked upregulation of c‐Jun phosphorylation in the 8‐week high IOP‐exposed mice group with a 2.34 ± 0.16‐fold increase compared to the uninjured (sham, normal IOP) control group (Figure 5A). Mice treated with siponimod showed a significantly reduced c‐Jun activation in the S1PR1^+/+^ control mice (*p* < .001) in high IOP conditions. S1PR1 deletion in RGCs abolished these protective effects of siponimod on the suppression of c‐Jun activation in glaucomatous conditions (Figure 5B). Significantly increased levels of c‐Jun activation were found in the Neu‐S1PR1^−/−^ mice compared to the S1PR1^+/+^ control mice group (*p* < .01) with the siponimod treatment in high IOP conditions.

We next evaluated the expression of proapoptotic Bim, which plays an important role in RGC development and degeneration in optic nerve injury conditions.^50^ Upregulation of Bim has been shown to be dependent on the c‐Jun activation.^51^ Immunofluorescence staining of retinal sections revealed a drastic increase in Bim expression in the RGC layer in high IOP conditions, and this upregulation was markedly reduced in S1PR1^+/+^ control mice treated with siponimod (Figure 5C). Immunoblotting analysis of retina lysates showed a significant decrease in Bim expression with siponimod treatment in high IOP conditions in S1PR1^+/+^ control mice, consistent with the immunofluorescence results. Mice subjected to S1PR1 deletion in RGCs failed to show decreased levels of Bim expression with the siponimod treatment. In immunoblot analysis, a significantly higher level of Bim expression was found in Neu‐S1PR1^−/−^ mice group compared to the control S1PR1^+/+^ mice group treated with siponimod (*p* < .01) in glaucomatous injury (Figure 5D,E). These results suggested that S1PR1 signaling in RGCs induces the neuroprotective effects by suppressing the c‐Jun/Bim proapoptotic activation via Akt/Erk pathway.

### 3.5 S1PR1‐mediated activation of Akt upregulates Bad phosphorylation in RGCs

**FIGURE 6:**
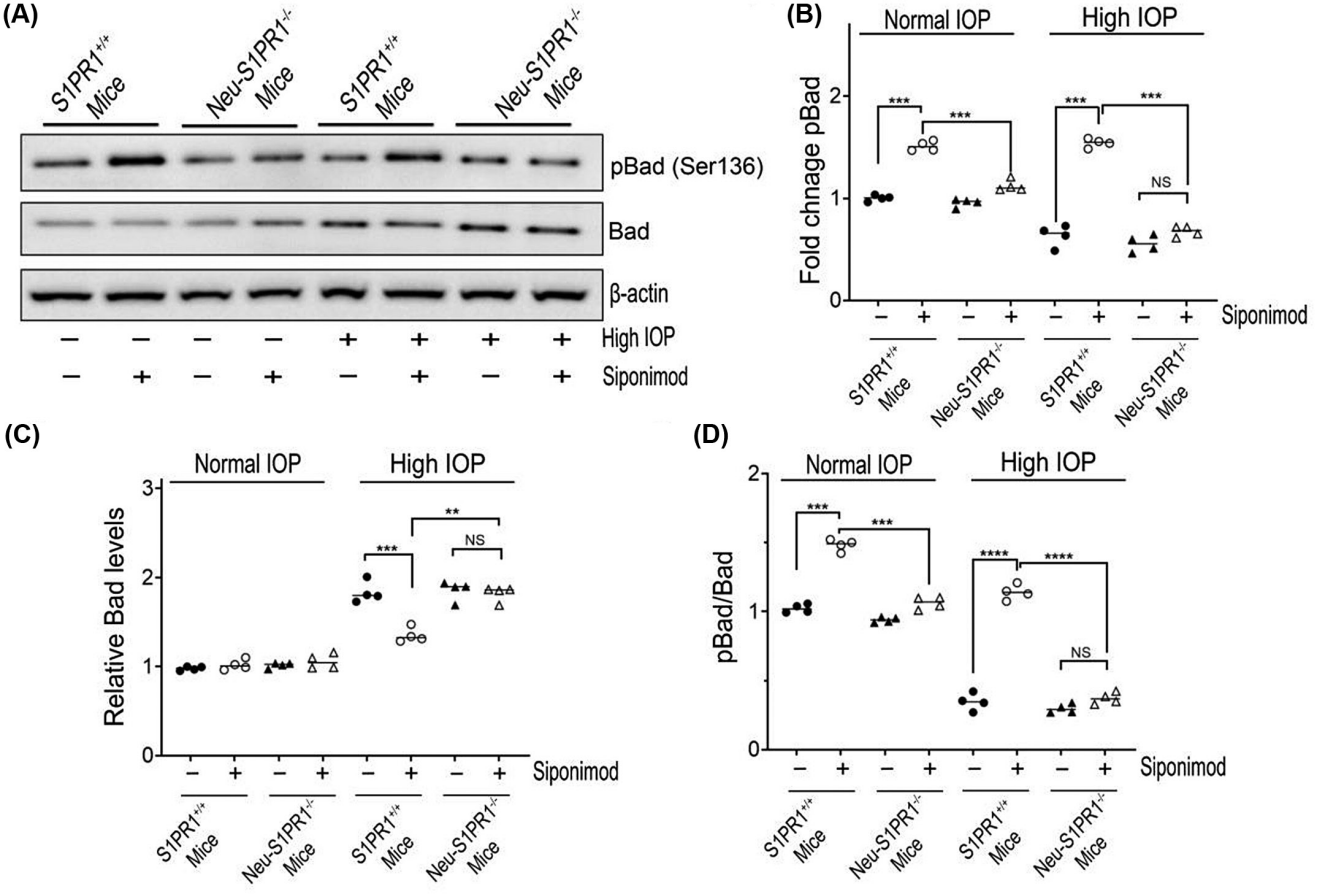
Activation of S1PR1 modulates the apoptotic pathway through upregulation of Bad phosphorylation (Ser 136) in experimental glaucomatous conditions. (A) Western blot analysis for Bad expression and phosphorylated levels of Bad (representative immunoblots) in the retinal samples from control S1PR1^+/+^ mice and Neu‐S1PR1^−/−^ (AAV‐GFP‐Cre) mice groups under normal and 8 weeks of high IOP conditions with/without siponimod treatment. (B) Densitometric quantitative analysis of blot densities (normalized to β‐actin) for phosphorylated Bad (Ser 136) and (C) the relative levels of Bad. (D) Decreased ratio of pBad/Bad in elevated IOP conditions is resorted to significant extent with siponimod treatment in control S1PR1^+/+^ mice and the deletion of S1PR1 in RGCs reduced the pBad/Bad ratios (**p < .01, ***p < .001, ****p < .0001 one‐way ANOVA analysis with Tukey's multiple comparisons test, *n* = 4 per group).

Bad is a proapoptotic Bcl‐2 family protein, that plays a critical role in promoting the apoptotic cascade in RGCs, and its activity is also regulated by phosphorylation.^52^, ^53^ Bad phosphorylation is critical for its action and dephosphorylated Bad can form a complex with antiapoptotic Bcl‐2 or Bcl‐xL at the mitochondrial membrane, leading to cytochrome *c* release and caspase activation to promote apoptotic cell death. Akt‐induced phosphorylation of Bad at Ser136 reduces its binding activity to bind to Bcl‐2 or Bcl‐xL.^54^, ^55^ To investigate whether the S1PR1 signaling‐mediated Akt activation regulates the Bad activity, we evaluated the retina lysates for Bad phosphorylation levels. Immunoblot analysis revealed an increased levels of Bad and decrease in Bad (Ser 136) phosphorylation in high IOP conditions (Figure 6A–C). Densitometric quantitative analysis showed a significant reduction of 2.81 ± 0.26‐fold in the pBad/Bad ratio in glaucomatous injury conditions compared to uninjured conditions (Figure 6D). The S1PR1^+/+^ control mice group treated with siponimod restored the Bad expression levels and significantly upregulated the pBad/Bad ratio (*p* < .0001). This positive effect of siponimod treatment on pBad levels was attenuated in the Neu‐S1PR1^−/−^ mice group. Deletion of S1PR1 in RGCs significantly reduced the pBad/Bad level compared to the control S1PR1^+/+^ mice group treated with siponimod (*p* < .0001) in high IOP‐induced glaucomatous conditions. These results suggest that S1PR1 activation in RGCs induces protective antiapoptotic effects through Bad phosphorylation through Akt activation.

## 4 DISCUSSION

S1PRs are G protein‐coupled receptors, composed of five subtypes, named S1P1‐5 receptors, and expressed by a wide range of cells in different organs including the heart, stomach, liver, brain, and retina.^37^, ^56^ Within the CNS, S1PRs are expressed in neurons, oligodendrocytes, astrocytes, and microglia and play important role in neurogenesis, neuronal development, myelination, and neuroinflammation.^36^ Modulation of S1PRs has been shown to have beneficial effects in animal models of neurodegenerative diseases, making it a promising therapeutic target.^57^ Most abundantly expressed S1PR1 in CNS is shown to regulate distinct survival intracellular signalings in neurons.^58^, ^59^, ^60^, ^61^ In this study, we have identified the mechanism of neuronal S1PR1 signaling that contributes to the RGCs survival in experimental glaucomatous injury.

S1PR1 in the CNS is recognized as a potential therapeutic target in several brain diseases because of its signaling involved in multiple cellular and pathophysiological processes.^56^ Fingolimod and siponimod are S1P structural analogs, that are approved drugs for the treatment of multiple sclerosis (MS).^21^, ^62^ Phosphorylated fingolimod binds to four S1PRs, S1P1, S1P3, S1P4, and S1PR5 receptors. On the other hand, siponimod selectively modulates S1P1 and S1P5 receptors.^23^ Presently, the widely accepted mechanism of action of both these drugs in MS patients is through immunosuppressive action by modulating S1PRs in the lymphocytes and subsequent inhibition of neuroinflammation. Fingolimod and siponimod can cross the blood–brain barrier and affect S1PR signaling in the CNS cells.^63^ In addition to anti‐inflammatory effects, the drugs have been reported to induce beneficial effects against stroke,^64^ Parkinson's disease (PD),^59^ and Alzheimer's (AD) degenerative changes.^16^, ^65^ Siponimod treatment has been shown to reduce disability progression in secondary progressive MS patients.^21^ This suggests the drug might exert direct neuroprotective effects because progressive MS is caused primarily by local CNS degenerative mechanisms.^66^ Our recent previous study identified that siponimod treatment exhibited neuroprotective effects on the mouse visual system through neuronal S1PR1 independent of peripheral immunomodulatory effects.^26^

To define the role and signaling mechanism of S1PR1 in RGCs degeneration in glaucomatous injury, we generated neuron‐specific deletion in S1PR1^*flox/flox*^ mice. S1PR1 is expressed by various types of neurons in the CNS and its deletion was reported to be embryonically lethal.^39^, ^56^ Neuron‐specific ablation of S1PR1 was achieved by intravenous administration of engineered AAV‐Cre recombinase constructs under Syn1 promoter into S1PR1^*flox/flox*^ adult mice that efficiently transduced the neuronal cells. In normal conditions, deletion of neuronal S1PR1 did not affect RGCs apoptotic changes measured with the TUNEL staining and western blot analysis of apoptotic‐related proteins, Bax, Bcl2, and activated caspase 3. However, the apoptotic changes in the GCL were significantly increased upon S1PR1 deletion in RGCs in high IOP conditions. Further, the protective effects of siponimod against apoptotic changes were significantly reduced in mice deleted for S1PR1 expression in RGCs in high IOP conditions. An increased expression of Bax and cleaved caspase 3, and a decreased expression of antiapoptotic Bcl2 proteins observed in high IOP conditions were restored to a significant extent with siponimod treatment in the S1PR1^+/+^ control mice group. These protective antiapoptotic effects of siponimod were significantly diminished in neuronal S1PR1‐deleted mice that correlated with the higher number of TUNEL‐positive cells. Previously, the S1P‐S1PR1 axis was demonstrated to play a vital role in neurogenesis, differentiation, and apoptosis, and its deletion caused increased embryonic neuronal death.^39^, ^67^ S1PR1 plays an important regulatory role in RGCs survival and axonal growth in acute optic nerve trauma conditions.^68^, ^69^ S1PR1‐mediated modulation of mTOR‐pS6 signaling cascade induced cell survival and silencing of S1PR1 in RGCs enhanced ON injury‐induced cell death and inhibited axonal growth.^68^ S1PR1 activation upregulates PI3K/Akt singling^15^ and the mTOR activation by Akt has been demonstrated to induce neuroprotective and axonal regeneration effects.^70^ This study further substantiates the neuroprotective properties of neuronal S1PR1 through antiapoptotic effects against RGCs degeneration in high IOP‐induced glaucoma conditions.

Akt and Erk1/2 are the key altered signaling pathways involved in the RGC apoptosis in experimental models of glaucoma and ON injury.^42^, ^71^, ^72^ These signal transduction pathways are also involved in the survival of neurons in different degenerative conditions.^18^ Activation of S1PR1 by S1P or fingolimod has been shown to upregulate Akt and Erk1/2 survival pathways through Gi protein.^15^ Our results showed significant downregulation of Akt and Erk1/2 phosphorylation levels in high IOP conditions. Siponimod‐treated mice demonstrated significantly higher levels of Akt, and Erk1/2 phosphorylation and these effects were reduced with neuronal deletion of S1PR1. These results indicated that S1PR1 in RGCs upregulates protective Akt and Erk1/2 that could promote downstream antiapoptotic effects against RGC degeneration in glaucoma injury. We next evaluated the downstream signaling events of Akt/Erk activation that protected RGCs against apoptosis. The transcription factor c‐Jun belongs to the JNK pathway, and its activation has been demonstrated in glaucoma and other neurodegenerative diseases.^43^, ^46^ The phosphorylation of c‐Jun on Ser63/73 by JNK promotes neuronal apoptosis by upregulating the expression of Bcl‐2 family members such as Bim, Puma, and Bmf.^51^, ^73^ Activation of the Akt/Erk pathway has been shown to negatively regulate apoptotic activation of JNK/c‐Jun to promote cell survival.^43^, ^48^, ^49^ In high IOP conditions, we observed increased phosphorylation of c‐Jun, and this activation was significantly reduced with siponimod treatment in the S1PR1^+/+^ control mice group. In neuronal S1PR1‐deleted mice, siponimod failed to suppress c‐Jun activation in glaucomatous injury. We next analyzed c‐Jun‐mediated transcriptional induction of Bim. Proapoptotic Bim is a Bcl‐2 family member that is known to induce Bax activation and play a critical role in RGCs apoptotic cell death.^51^ Our results from immunofluorescence and western blot analysis showed a marked upregulation of Bim in glaucomatous injury and this upregulation was significantly diminished with siponimod treatment. Our data showed Bim expression was enhanced in association with the activation of c‐Jun in neuronal S1PR1‐deleted mice. Together, these results suggest that activation of Akt/Erk pathways downstream of S1PR1 induces antiapoptotic effects via c‐Jun/Bim axis.

Further, in another set of results, we identified that S1PR1‐mediated activation of Akt regulates Bad phosphorylation. Proapoptotic factor Bad in the dephosphorylated form promotes the apoptotic cascade by binding to antiapoptotic proteins Bcl‐X_L_ and Bcl‐2, inhibiting their functions.^55^ Phosphorylation of Bad at Ser136 by Akt has been shown to promote cell survival by impairing its binding ability with Bcl‐X_L_ and Bcl‐2.^54^, ^58^ Our results showed a drastically decreased pBad/Bad ratio in glaucomatous retinas, and siponimod‐treated mice showed significantly enhanced pBad/Bad levels. These increased levels of phosphorylated Bad were reduced in neuronal S1PR1‐deleted mice with siponinod treatment. These results indicate that S1PR1‐mediated activation of Akt is associated with the phosphorylation of Bad that promotes neuronal survival. Phosphorylation of Bad at Ser138 by activated Akt was demonstrated to be neuroprotective against neuronal damages.^19^, ^74^

Neuroprotective strategies aim to maintain ganglion cell function and stimulate axonal regeneration by inducing intrinsic and extrinsic pathways involved in neuroprotection. Intrinsic activation of PI3K/Akt and Erk cascades by S1PR1 and neurotrophic factors including brain‐derived neurotrophic factor (BDNF) and ciliary neurotrophic factor (CNTF) were shown to induce RGC survival and axonal regeneration via glycogen synthase kinase (GSK3β), mTOR, and antiapoptotic pathways.^10^, ^68^, ^75^ CTNF exerted potent axonal regenerative effects via activated Stat3 and regulates the S1PR1 expression in ON injury.^69^ S1PR1 downregulation repressed the axonal sprouting and increased RGCs loss in ON injury conditions, suggesting the neuroprotective roles of S1PR1 in the retina.^68^ In this study, S1PR1 activation with siponimod inhibited the proapoptotic cJun/Bim cascade in experimental glaucoma and cJun N‐terminal kinase inhibitors have previously shown neuroprotection for RGCs in the retina.^76^

In the mammalian retinal tissue, S1PR1, S1PR2, and S1PR3 are the prominently expressed receptor subtypes.^12^ S1PR2 simulation in RGCs has been shown to induce deleterious effects that inhibited axonal growth via Rho‐A activation.^68^ S1PR2 has also been shown as a receptor for Nogo, which is a potent inhibitor for neuronal growth and plasticity in the CNS.^77^ In the CNS, S1PR5 is specifically expressed by endothelial cells of the BBB and is a dominant S1P receptor subtype expressed by oligodendrocytes.^13^, ^56^ S1PR5 has minimal expression in the retinal tissue and is restricted to RPE and endothelium of the BBB.^12^, ^78^ Siponimod which also binds to S1PR5 did not induce any significant extent of protective effects on the RGC survival pathway in the mice specifically deleted for S1PR1 in RGCs suggesting that protective effects are mainly mediated by S1PR1 in glaucoma. Although axonal injury is the key insult in glaucoma that causes degenerative changes in the inner retina, inflammatory responses of activated microglia, and reactive gliosis are also implicated in the pathogenesis. Activated glial cells observed in various retinal layers are also thought to mediate pro‐inflammatory changes in glaucoma progression.^79^ S1PRs also regulate systemic inflammatory processes^10^, ^36^ and their implications in regulating ocular microenvironment remain to be elucidated.

**FIGURE 7:**
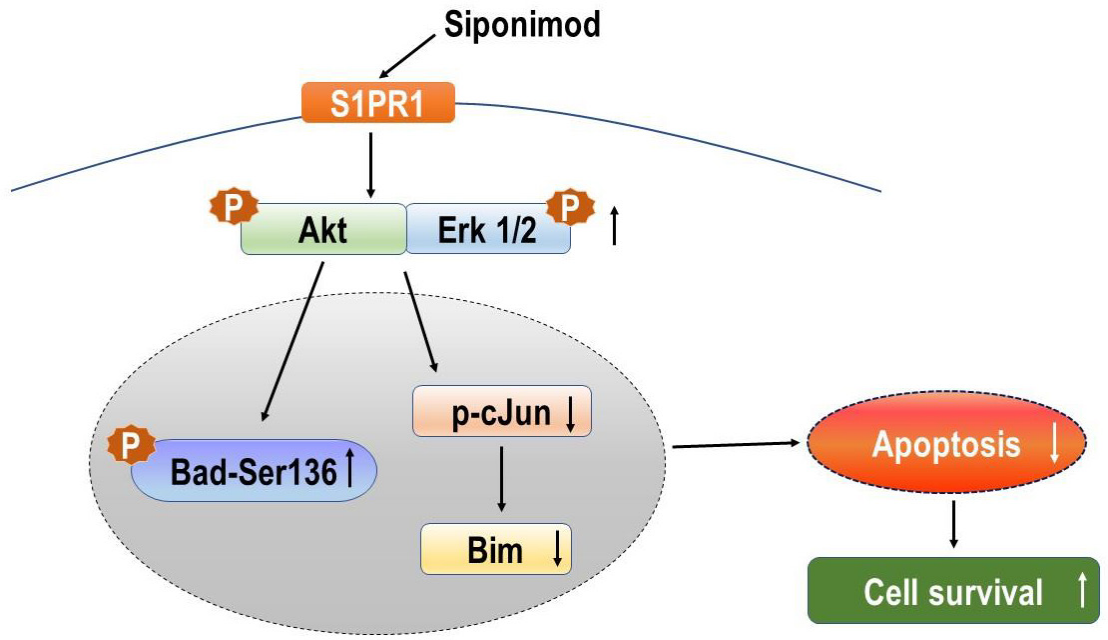
Proposed mode of S1PR1‐mediated antiapoptotic signaling in retinal ganglion cells in experimental glaucoma. Modulation of S1PR1 by siponimod activates Akt/Erk pathway that resulted in suppression of apoptotic pathway via cJun/Bim cascade and phosphorylation of Bad (Ser 136).

In summary, this study demonstrated that S1PR1 signaling in RGCs exerts antiapoptotic effects in an experimental glaucoma model. Our results provide the first evidence that S1PR1‐mediated activation of Akt/Erk induces antiapoptotic events through suppression of the c‐Jun/Bim cascade and Bad phosphorylation to promote RGCs survival in glaucoma. Further, this study establishes that the protective effects of siponimod are mediated through neuronal S1PR1 by promoting key intracellular Akt/Erk survival signaling cascades (Figure 7).

## AUTHOR CONTRIBUTIONS

Devaraj Basavarajappa, Vivek Gupta, Mehdi Mirzaei, Alexander Klistorner, and Stuart L. Graham designed research; Devaraj Basavarajappa, Roshana Vander Wall, Nitin Chitranshi, Viswanthram Palanivel, and Seyed Shahab Oddin Mirshahvaladi performed experiments and acquitted the data; Devaraj Basavarajappa, Vivek Gupta, Yuyi You, and Veer Gupta analyzed data; All authors helped to write the manuscript.

## DISCLOSURES

The authors declare that they have no competing interests.

## Supporting information


Figure S1
Figure S2
Figure S3

## Data Availability

The authors declare that all the relevant data, associated protocols, and materials supporting the findings of this study are present in the article.
