# Supplementary material for: S1PR1 signaling attenuates apoptosis of retinal ganglion cells via modulation of cJun/Bim cascade and Bad phosphorylation in a mouse model of glaucoma
Source: FASEB J. 2022 Dec 15;37(1):e22710. doi: 10.1096/fj.202201346R (PMC13281836; doi:10.1096/fj.202201346R)
Supplement: Supplementary file 1 — Figure S1 Figure S2 Figure S3 [file FSB2-37-e22710-s001.docx]

**Supplementary Figures for**

**S1PR1 signaling attenuates apoptosis of retinal ganglion cells via modulation of cJun/Bim cascade and Bad phosphorylation in a mouse model of glaucoma**

**This file includes:**

Supplemental **Figures S1-S3**

**
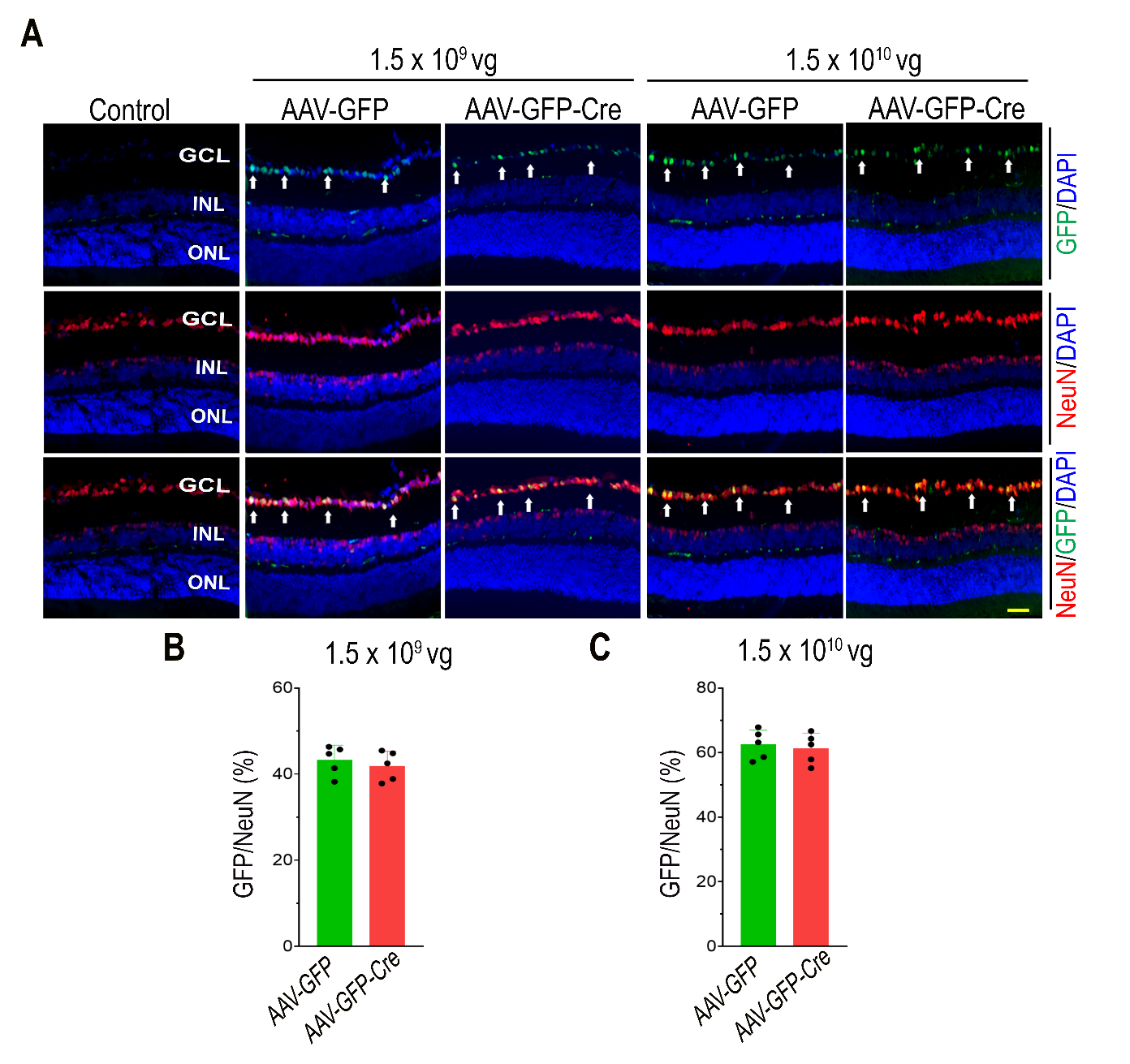
**

**Figure S1. Dose-dependent transduction efficiency of AAV-PHP.eB in S1PR1*^flox/flox^* transgenic mice retinas** (**A**) Immunofluorescence images of the eye sections (representative; GCL, ganglion cell layer; INL, inner nuclear layer; ONL, outer nuclear layer) stained with GFP (green) and NeuN (red) showing the infection of RGCs with AAVs delivered via tail vein injection (arrows indicate the expression and colocalization of GFP expression in RGCs in the GCL, Scale bar=50 µM). (**B**) Transduction efficiency (GFP^+^ cells/total NeuN cells) of the viral vector was measured after two months of AAV delivery for the mice that received 1.5 × 10^9^ vg and (**C**) 1.5 × 10^10^ vg (n=5 per group).

**
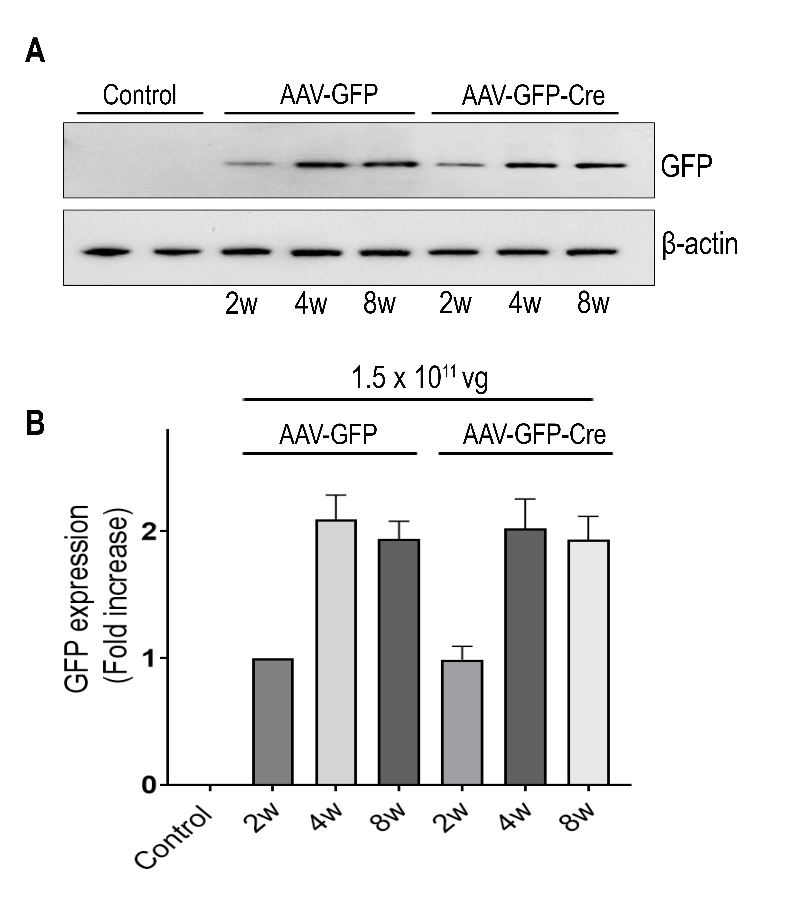
**

**Figure S2. Time course of GFP expression changes in the S1PR1*^flox/flox^* mice retinas transduced with AAV-PHP.eB. (A**) Western blot Analysis of the retinal tissues for GFP expression at different time points (weeks) following delivery of 1.5 × 10^11^ vg AAVs via tail vein injections. (**B**) Densitometric quantification of western blot band intensities for GFP expression after normalizing to β-actin (n=3 per group).

**
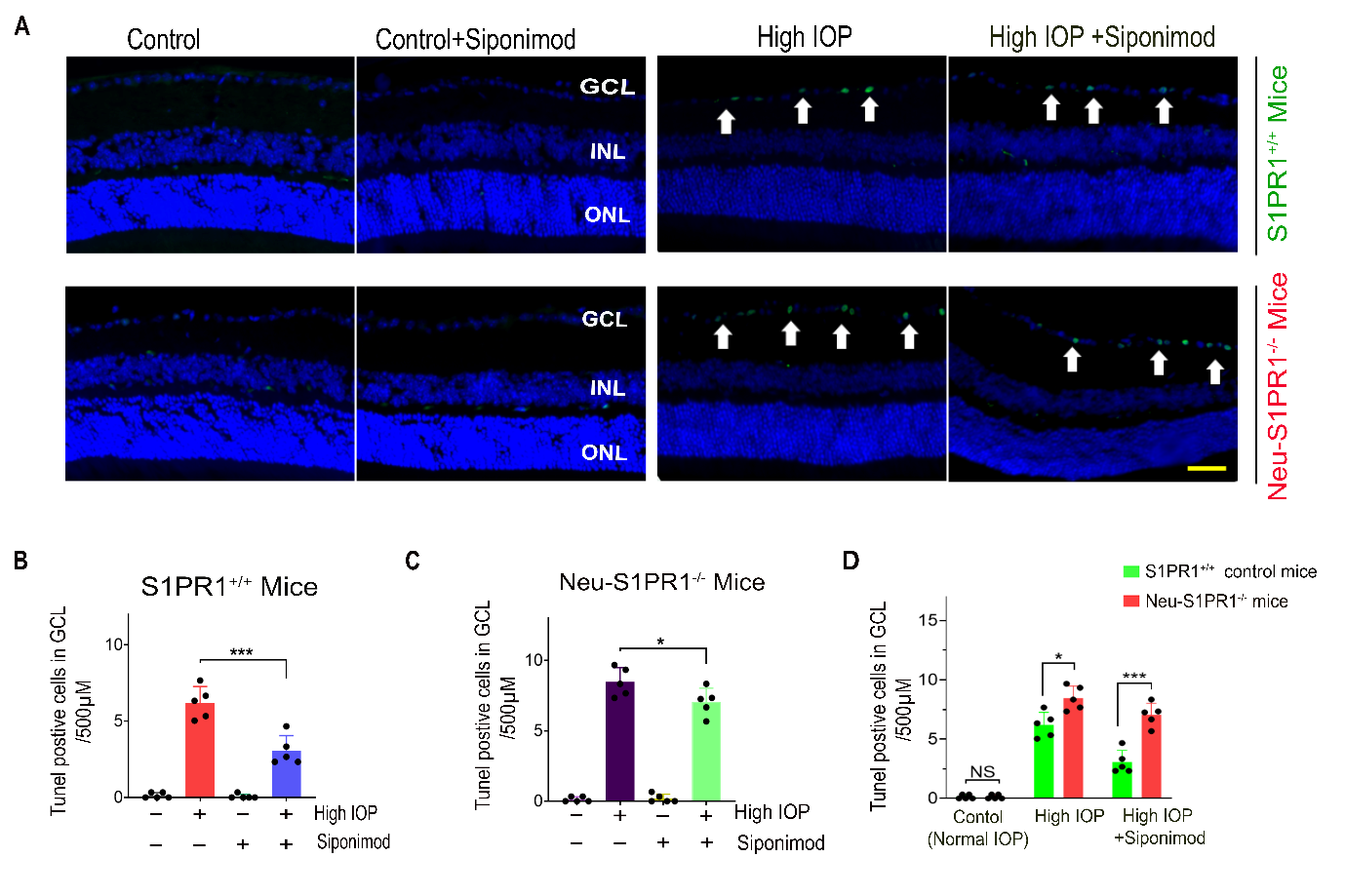
Figure S3. Analysis of apoptotic changes in the ganglion cell layer (GCL) assessed in 600-1200 μm region from the edge of the optic disk in retinal sections** (**A**) Representative images of retinal sections from the TUNEL staining (arrows indicate changes in the TUNEL staining in the GCL) after 8 weeks of elevated IOP induction in different mice groups (TUNEL staining (green) and nuclear staining DAPI (blue), Scale bar=50 µM; GCL, ganglion cell layer; INL, inner nuclear layer; ONL, outer nuclear layer). (**B**) Quantification of TUNEL-positive cells in the GCL of retinas from the S1PR1^+/+^ control mice group and (**C**) Neu-S1PR1^-/-^ mice group. (**D**) Comparison of the TUNEL-positive cells in the GCL among different mice groups showed S1PR1 deletion in RGCs significantly increased the apoptotic cells and diminished the protective effects of siponimod in elevated IOP conditions (NS, not significant, **P*<0.05, ****P*<0.001, one-way ANOVA analysis with Tukey’s multiple comparisons test, n=5 per group).
